# Salvage resection after immunotherapy for giant undifferentiated pleomorphic sarcoma of the chest wall

**DOI:** 10.1093/jscr/rjaf747

**Published:** 2025-10-27

**Authors:** Akifumi Nakamura, Ayumi Kuroda, Masaki Hashimoto, Nobuyuki Kondo, Takashi Yamasaki, Kozo Kuribayashi, Takashi Kijima, Seiki Hasegawa, Soichiro Funaki

**Affiliations:** Division of Thoracic Surgery, Department of Surgery, Hyogo Medical University, 1-1 Mukogawa-cho, Nishinomiya, Hyogo 663-8501, Japan; Division of Thoracic Surgery, Department of Surgery, Hyogo Medical University, 1-1 Mukogawa-cho, Nishinomiya, Hyogo 663-8501, Japan; Division of Thoracic Surgery, Department of Surgery, Hyogo Medical University, 1-1 Mukogawa-cho, Nishinomiya, Hyogo 663-8501, Japan; Division of Thoracic Surgery, Department of Surgery, Hyogo Medical University, 1-1 Mukogawa-cho, Nishinomiya, Hyogo 663-8501, Japan; Department of Surgical Pathology, Hyogo Medical University, 1-1 Mukogawa-cho, Nishinomiya, Hyogo 663-8501, Japan; Department of Respiratory Medicine and Hematology, Hyogo Medical University, 1-1 Mukogawa-cho, Nishinomiya, Hyogo 663-8501, Japan; Department of Respiratory Medicine and Hematology, Hyogo Medical University, 1-1 Mukogawa-cho, Nishinomiya, Hyogo 663-8501, Japan; Department of Thoracic Surgery, Takarazuka City Hospital, 4-5-1, Kohama, Takarazuka, Hyogo 665-0827, Japan; Division of Thoracic Surgery, Department of Surgery, Hyogo Medical University, 1-1 Mukogawa-cho, Nishinomiya, Hyogo 663-8501, Japan

**Keywords:** undifferentiated pleomorphic sarcoma, salvage surgery, nivolumab, ipilimumab

## Abstract

We present a rare case of primary undifferentiated pleomorphic sarcoma (UPS) of the chest wall in a 45-year-old male, initially diagnosed as sarcomatoid pleural mesothelioma via computed tomography (CT)-guided biopsy. CT revealed a large tumor measuring 13 × 12 cm occupying the left thoracic cavity, with extensive invasion into the left first through fifth ribs and the adjacent lung parenchyma. The patient was referred to our hospital and treated with a combination of nivolumab and ipilimumab, resulting in marked tumor regression. Upon reevaluation, the diagnosis was revised to sarcoma, and the patient subsequently underwent salvage surgery, including resection of the left upper lung lobe and first through fifth ribs. Histopathology confirmed tumor mutational burden-high UPS. The patient has remained disease-free for two and a half years postoperatively. This case demonstrates the potential efficacy of immune checkpoint inhibitors in multimodal treatment for select cases of chest wall UPS.

## Introduction

Undifferentiated pleomorphic sarcoma (UPS), previously known as malignant fibrous histiocytoma, is an aggressive soft tissue sarcoma that lacks a distinct histological classification [1]. Here, we report a rare case of primary chest wall UPS where significant immunotherapy-induced tumor regression enabled complete surgical resection.

## Case report

**Figure 1:**
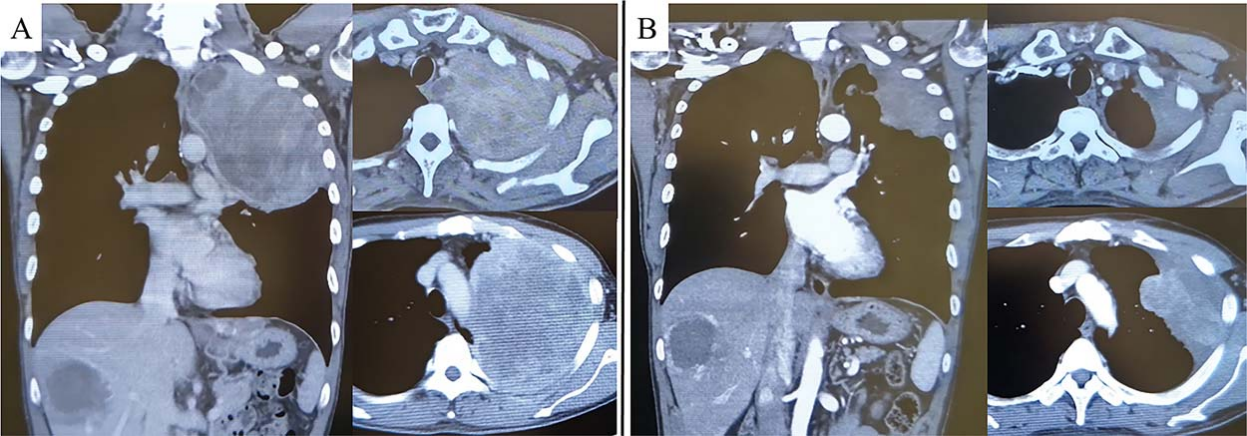
Pre-treatment CT revealed a large mass exceeding 13 cm in the left lung apex, with invasion into the first through fifth ribs (A). Following treatment with nivolumab with ipilimumab, the tumor showed marked shrinkage (B).

**Figure 2:**
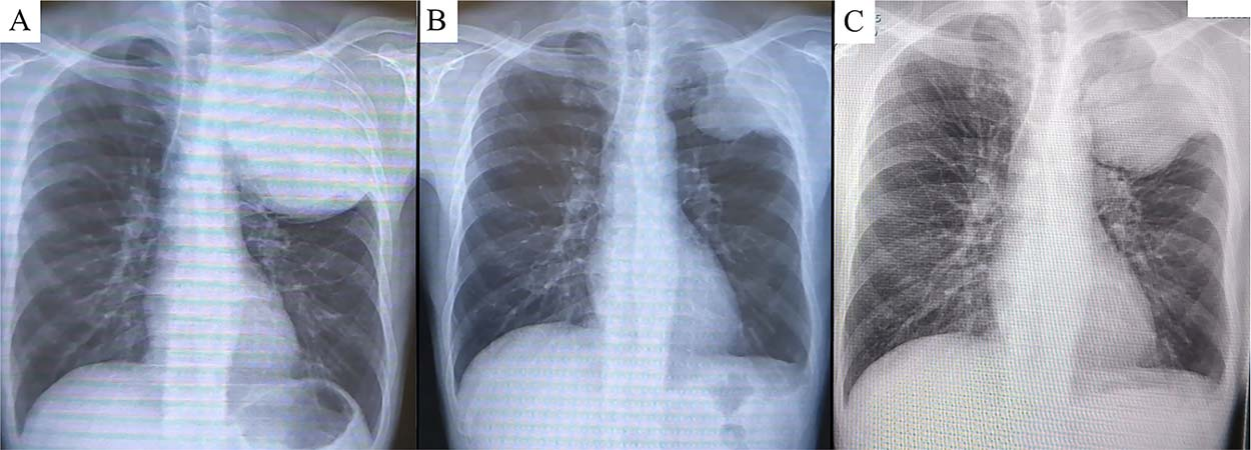
Chest X-ray prior to treatment revealed a large mass extending from the left lung apex to the upper lung field (A), which markedly decreased in size following immunotherapy (B). However, during the 4-week interval between the end of immunotherapy and surgery, the tumor demonstrated regrowth (C).

A 45-year-old male presented with left anterior chest pain. A chest X-ray revealed a mass in the left lung. Computed tomography (CT) showed a large tumor measuring 13 × 12 cm occupying the left thoracic cavity, with extensive invasion into the left first through fifth ribs and lung parenchyma. A CT-guided biopsy initially led to a diagnosis of sarcomatoid pleural mesothelioma, and the patient was referred to our hospital. The patient exhibited tumor-associated fever and elevated inflammatory markers. Treatment with nivolumab plus ipilimumab was initiated promptly. A re-evaluation of the biopsy specimen revealed proliferations of short spindle-shaped tumor cells. However, BAP1 was retained, MTAP status was inconclusive, and no CDKN2A/CEP9 probe deletion was observed, ruling out pleural mesothelioma. The final biopsy diagnosis was sarcoma. After four cycles of nivolumab plus ipilimumab, the tumor size significantly decreased (from 139 × 122 mm to 97 × 76 mm), leading to the decision to proceed with salvage surgery (Fig. 1A and B). Despite a 4-week interval between the final immunotherapy dose and the surgery, tumor regrowth was observed during this period (Fig. 2A–C).

**Figure 3:**
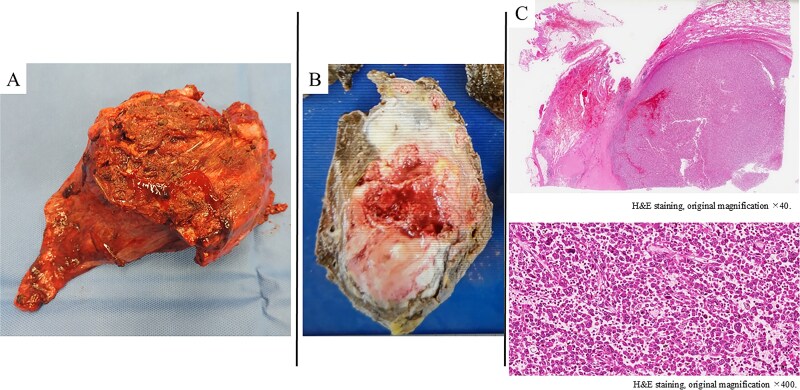
A poorly defined, grayish-white mass measuring 100 × 50 mm, observed macroscopically in the chest wall and left upper lobe (A, B). Histologically (H&E staining), the tumor showed solid proliferation of pleomorphic tumor cells accompanied by fibrosis, hyalinization, and infiltration of inflammatory cells (C).

The surgery was initiated with a Paulson incision. A thoracotomy through the fifth intercostal space revealed extensive adhesions throughout the thoracic cavity, necessitating adhesiolysis. The tumor had invaded the region cranial to the fifth rib, requiring a combined resection of the first through fifth ribs—maintaining a safe margin from the tumor. The tumor had also extensively invaded the left upper lobe of the lung, leading to a left upper lobectomy and complete tumor resection. The chest wall defect was reconstructed using Prolene mesh. The operation time was 252 min, and blood loss was 490 ml. The postoperative course was uneventful, and the patient was discharged in good condition on postoperative day seven. Macroscopically, a solid, gray-white tumor measuring 100 × 50 mm was observed centered on the chest wall (Fig. 3A and B). Amid a background of fibrosis, hyalinization, and inflammatory cells, tumor cells exhibiting pleomorphism and mitotic activity were proliferating (Fig. 3C). Areas of the tumor demonstrated dense lymphocytic infiltration and regions with fibrosis and hyalinization without viable tumor cells. The Ki-67 labeling index was 70%. Programmed Death-Ligand 1 (PD-L1) expression was not assessed. The surgical margins were histologically negative, confirming an R0 resection. Mesothelioma and lung cancer markers were negative. The final diagnosis was UPS. Additionally, genomic profiling using FoundationOne CDx revealed a tumor mutational burden-high result. No targetable genetic alterations were identified. The patient has remained disease-free for two and a half years postoperatively without adjuvant chemotherapy.

## Discussion

UPS accounts for 10%–15% of soft tissue sarcomas and is most common in adults aged 50 to 70 years [2]. UPS primarily arises in the extremities, particularly the thighs, and in the retroperitoneum and trunk [1]. Primary occurrence in the chest wall is exceedingly rare [1]. A systematic review of primary chest wall UPS evaluated 32 patients and found a higher prevalence in females, with 75% of tumors exceeding 5 cm in size [1]. Surgical resection is the primary treatment modality, with a critical margin distance of 1.5 cm. While radiotherapy and chemotherapy are used as adjunctive therapies, their application did not correlate with the site of recurrence. However, radiotherapy was shown to improve progression-free survival (PFS). Patients with deeper and larger tumors (typically >5 cm) have a worse prognosis.

Chemotherapy is commonly used in high-grade UPS treatment, with anthracycline-based medications, ifosfamide, and gemcitabine plus docetaxel being the primary agents [3]. Although the efficacy of these treatments remains debated, growing evidence suggests the potential role of adjuvant chemotherapy in reducing distant recurrence [1].

Although not currently approved by the US Food and Drug Administration, several recent reports have described the use of immunotherapy for UPS [4, 5]. A Phase II trial of neoadjuvant checkpoint blockade therapy for patients with surgically resectable UPS and dedifferentiated liposarcoma (NCT03307616) was conducted at MD Anderson Cancer Center [4]. In the trial, 10 patients with resectable UPS received neoadjuvant therapy with nivolumab or ipilimumab plus nivolumab in combination with radiation therapy. The primary endpoint, pathological response rate, was 89%. In the interim report presented at American Society of Clinical Oncology 2022, with a follow-up period of over 2 years, the median PFS in patients with UPS was not reached [6]. The 1-year and 2-year PFS rates were 80% and 70%, respectively, with particularly favorable outcomes in the nivolumab plus ipilimumab group (1-year and 2-year PFS rates: 100%). However, the nivolumab monotherapy group had a 1-year PFS rate of 67% and a 2-year PFS rate of 50%. A multicenter phase II study evaluated the safety and efficacy of pembrolizumab in patients with advanced soft tissue sarcoma and osteosarcoma (SARC028) [5]. The primary endpoint, the objective response rate, was 40% (4 out of 10 patients) in patients with UPS. The secondary endpoints included a median PFS of 49 weeks, while median overall survival was not reached. These results suggest that immune checkpoint blockade induces durable responses and demonstrates meaningful clinical activity in patients with this subtype. PD-L1 expression has been associated with T-cell infiltration in UPS, suggesting that UPS may fit the model of an inflamed tumor, potentially explaining the efficacy of single-agent anti-PD-1 antibodies in this disease [5].

In this case, the patient was initially diagnosed with sarcomatoid pleural mesothelioma and was treated with nivolumab plus ipilimumab. The tumor demonstrated remarkable shrinkage, prompting surgical resection, which was successfully performed. The final diagnosis was an exceedingly rare primary UPS of the chest wall. Pathological examination demonstrated fibrosis, hyalinization, and prominent lymphocytic infiltration within the tumor stroma, with areas devoid of viable tumor cells. These histological findings may reflect immune-mediated tumor regression induced by immune checkpoint inhibition, although a definitive causal relationship cannot be established. Consistent with recent reports, this case suggests the potential efficacy of immunotherapy for this rare malignancy. Nevertheless, given the limited clinical evidence and heterogeneous treatment responses, further investigations are warranted to clarify their therapeutic efficacy and to identify predictive biomarkers for response in this rare malignancy.

## References

[1] Bennett C, Bharadwaj S, Arndt A, et al. A systematic review of undifferentiated pleomorphic sarcoma of the chest wall. Chin Clin Oncol 2023;12:66. 10.21037/cco-23-71 38073309

[2] Sbaraglia M, Dei Tos AP. The pathology of soft tissue sarcomas. Radiol Med 2019;124:266–81. 10.1007/s11547-018-0882-7 29948548

[3] Gronchi A, Ferrari S, Quagliuolo V, et al. Histotype-tailored neoadjuvant chemotherapy versus standard chemotherapy in patients with high-risk soft-tissue sarcomas (ISG-STS 1001): an international, open-label, randomised, controlled, phase 3, multicentre trial. Lancet Oncol 2017;18:812–22. 10.1016/S1470-2045(17)30334-0 28499583

[4] Keung EZ, Lazar AJ, Torres KE, et al. Phase II study of neoadjuvant checkpoint blockade in patients with surgically resectable undifferentiated pleomorphic sarcoma and dedifferentiated liposarcoma. BMC Cancer 2018;18:913. 10.1186/s12885-018-4829-0 30249211 PMC6154892

[5] Tawbi HA, Burgess M, Bolejack V, et al. Pembrolizumab in advanced soft-tissue sarcoma and bone sarcoma (SARC028): a multicentre, two-cohort, single-arm, open-label, phase 2 trial. Lancet Oncol 2017;18:1493–501. 10.1016/S1470-2045(17)30624-1 28988646 PMC7939029

[6] Keung EZ, Nassif EF, Lin HY, et al. Randomized phase II study of neoadjuvant checkpoint blockade for surgically resectable undifferentiated pleomorphic sarcoma (UPS) and dedifferentiated liposarcoma (DDLPS): survival results after 2 years of follow-up and intratumoral B-cell receptor (BCR) correlates. J Clin Oncol 2022;40:LBA11501.

